# Stigmasterol sensitizes endometrial cancer cells to chemotherapy by repressing Nrf2 signal pathway

**DOI:** 10.1186/s12935-020-01470-x

**Published:** 2020-10-06

**Authors:** Hong Liao, Dan Zhu, Mingzhu Bai, Huifen Chen, Shihuan Yan, Jing Yu, Huiting Zhu, Wenxin Zheng, Guorong Fan

**Affiliations:** 1 Department of Clinical Laboratory Medicine, Shanghai First Maternity and Infant Hospital, Tongji University School of Medicine, Shanghai, 200040 China; 2 The Graduate School, Tongji University School of Medicine, Shanghai, 200040 China; 3 College of Pharmacy, Guangxi Medical University, Nanning, 530021 China; 4 Department of Gynecology and Obstetrics, Shanghai General Hospital, Shanghai Jiaotong University, 100 Haining Road, Shanghai, 200080 China; 5 Department of Pathology, Shanghai First Maternity and Infant Hospital, Tongji University School of Medicine, Shanghai, 200040 China; 6 Department of Pathology, University of Texas Southwestern Medical Center, Dallas, TX 75390 USA; 7 Department of Obstetrics and Gynecology, University of Texas Southwestern Medical Center, Dallas, TX 75390 USA; 8 Department of Pharmacy, Shanghai General Hospital, Shanghai Jiaotong University, 100 Haining Road, Shanghai, 200080 China

**Keywords:** Nrf2, Chemoresistance, Hydroxymethylation, Stigmasterol, Endometrial cancer

## Abstract

**Background:**

Chemoresistance reduces the 5-year survival rate of endometrial cancer patient, which is the current major obstacle for cancer therapy. Increasing evidence state that Nrf2 contributes to chemoresistance in several kinds of cancer. However, its role in endometrial cancer cells remains unclarified.

**Methods:**

Immunohistochemistry staining was used to detect the expression of Nrf2 in normal patient and endometrial cancer patient. Stable transfection Ishikawa cell line with high level of Nrf2 was established to evaluate its role in chemoresistance. Dot blot assays were used to assess global hydroxymethylation level after stigmasterol treatment. Cellular growth profile was detected by CCK8 assay. Western blot was used to evaluate the changes of the target molecules after various treatments.

**Results:**

Nrf2 is overexpressed in endometrial cancer tissues compared with the normal endometrium. Overexpression of Nrf2 resulted in decrease sensitivity to cisplatin. In addition, stigmasterol has been identified as a novel Nrf2 inhibitor. It enhanced the sensitivity of endometrial cancer cells to cisplatin, and the underlying mechanism is that stigmasterol declines the Nrf2 protein level.

**Conclusions:**

Our findings identified stigmasterol as a new potential inhibitor of Nrf2 and highlight a critical role of stigmasterol in overcoming chemoresistance in endometrial cancer therapy.

## Background

Endometrial cancer is the main gynecological malignancy and most of cases occur in post-menopausal women [1–4]. In recent decade, it shows an increasing incidence rate, especially in developing country [3, 5]. Usually, surgery is the optimal therapy strategy for the patients with early-stage endometrial cancer, whereas chemotherapy is chosen to administrate the advanced and recurrent patients. As the first-line chemotherapeutics for endometrial cancer therapy, cisplatin and paclitaxel exhibit well-inhibition effect on cancer cell growth, however, chemoresistance is still a major obstacle in endometrial cancer therapy [6]. Therefore, it is urgent to find a biomarker of chemoresistance and develop a new and effective anticancer therapeutic regimen or strategy.

As a critical molecule of antioxidant response, Nrf2 has multiple roles in cancer development including chemoresistance, proliferation and anti-apoptosis [7–10]. In previous study, the bad effects of Nrf2 has been addressed, wang et al. demonstrated that high level of Nrf2 enhances chemoresistance, whereas knocking down Nrf2 sensitizes cancer cells to chemotherapy [11]. Consistent with these findings, it was showed that suppression of Nrf2 sensitizes ovarian cancer cells to doxorubicin and cisplatin [8]. Other study also found that xenografts derived from Nrf2-silenced lung cancer cells were more sensitive to carboplatin treatment [12, 13]. These data suggest that Nrf2 play an essential role in chemoresistance. However, how it involves in chemoresistance is not clear in endometrial cancer.

It has been found that many compounds are able to inhibit the expression of Nrf2 protein and its downstream target genes containing an enhancer sequence known as the antioxidant response element (ARE) [14–17]. Some of the target genes have the function to detoxification and removal of pharmacologic agents, which may contribute to chemoresistance [18–20]. Therefore, targeting Nrf2 and Nrf2-regulated downstream gene is a good strategy for cancer therapy. Zhang et al. first found that brusatol could enhance the chemotherapy sensitivity by facilitating the degradation of Nrf2 protein [21]. Consistent with this study, we also found that brusatol could enhance the metformin-induced inhibition effect on endometrial cancer [22]. In addition, other study also pointed out that metformin can decline the mRNA level and protein level in HepG2 cells, and the decline of Nrf2 is mediated by keap1-independent and AMPK-independent pathway [23]. Some of studies reported a number of Nrf2 inhibitors through high-throughput screening, such as dexamethasone, clobetasol propionate, wogonin, camptothecin [14, 15, 17, 24, 25]. Although these compounds are potential candidate inhibitors, the inhibition effect on Nrf2 expression is not very strong. It is far from the inhibition effect of brusatol on Nrf2 expression. Therefore, it is necessary to search more effective compound to enhance chemotherapy sensitivity by downregulation of Nrf2. Previous study mentioned that the extract from mangrove has the effect of antioxidant response and anti-inflammation, this prompts us to investigate whether some of the compound from this plant may have the inhibition effect on Nrf2 expression and Nrf2-induced biological function, such as chemoresistance. Therefore, the objective of this study is to clarify the molecular mechanisms how Nrf2 involves in chemoresistance in endometrial cancer and find some of the candidate inhibitors to overcome drug resistance, which would be relevant to development of new therapeutic strategies that would improve patient care.

## Materials and methods

### Cell culture

The endometrial cancer cell lines, Ishikawa and SPEC2, were used in this study. MDA-MB-231 is a breast cancer cell line. Ishikawa and MDA-MB-231 cell lines were purchased from the American Type Tissue Type Tissue Collection (Manassas, VA, USA), SPEC2 is kindly provided by Dr. Zheng W (University of Texas Southwestern Medical Center, Dallas, TX, USA) and maintained in our lab. These cell lines were cultured in Medium DMEM: F12 (1:1, GIBCO) with 100 µg/ml streptomycin (Life Technologies, Inc., Rockville, MD), 100 U/ml penicillin G and 10% fetal bovine serum (FBS; Gibco, Gaithersburg, MD, USA).

### IHC

Total 120 endometrial tissue samples, including 10 cases of normal endometrium, 20 cases of simple hyperplasia and 90 cases of endometrial cancer, were enrolled in this study. These samples were collected from the Department of Obstetrics and Gynecology of Shanghai First Maternity and Infant Hospital, Tongji University School of Medicine, China. Samples collected in compliance with institutional review board regulations from the Medical College, Tongji University, China.

IHC analysis of Nrf2 expression pattern was performed as previously described. Briefly, firstly samples were deparaffinized in xylene and rehydrated in a graded series of ethanol, subsequently, 3.0% hydrogen peroxide was used to block the endogenous peroxidase activity. Following antigen retrieval, incubation overnight with rabbit anti-human Nrf2 primary antibodies at 4 °C in a humid chamber, followed by 60-min incubation with biotinylated secondary antibody (Dako, Carpinteria, CA, USA). Omitted primary antibodies were used as negative controls. Expression of Nrf2 was assessed as previous described.

### RNA isolation and quantitative real time PCR

Total RNA was harvested with Trizol reagent. 1 µg of total RNA was reverse transcribed into cDNA using the reverse transcriptase kit (Qiagen company, Frankfurt, Federal Republic of Germany). The primers used to amplification of Nrf2 and GAPDH were listed Table 1. The water was served as negative control.

**Table 1:** Sequences of primers used for amplification of target genes

Gene	Primer nucleotide sequence
Nrf2	Forward: 5′-CACATCCAGTCAGAAACCAGTGG-3′
	Reverse: 5′-GGAATGTCTGCGCCAAAAGCTG-3′
GAPDH	Forward: 5′-AACGGATTTGGTCGTATTG-3′
	Reverse: 5′-GGAAGATGGTGATGGGATT-3′

### Western blot

The changes of the proteins were detected with western blot. The cells underwent indicated treatment were lysed and loaded to SDS–polyacrylamide gel, electrophoresed and transferred to PVDF membranes, then incubated primary antibodies (Nrf2, #12721, CST company; #ab62352, Abcam company) and second antibodies. After final wash with PBS, the bands was detected with chemiluminescence detection system (ECL detection kit; Pierce, Rockford, IL). Each experiment was performed at least three times.

### Drug treatment, cell proliferation and clonogenic assay

Endometrial cancer cells were treated with cisplatin, stigmasterol, or combine to both drugs with indicated doses. Cell proliferation pattern was monitored with CCK8 assay. A clonogenic assay was carried out referring previously study [26].

### Plasmid transfection

Tet1 plasmid was transfected into Ishikawa cells using Lipofectamine 3000 TM (Invitrogen) according to the manufacturer’s protocol. The transfection efficiency was confirmed by western blot.

### Establishment of stable cell lines

To identify the role of Nrf2 in chemotherapy resistance, the stable cell lines with high levels of Nrf2 or Keap1 were established. Ishikawa- and Spece2 derived stable cell lines, with incorporation of Nrf2 or Keap1, were established using lentivirus system as described previously. Stable Ishikawa and spece2 cells were continuously cultured in medium containing 1.5 μg/ml puromycin (sigma). The efficiency of transfection was determined with western blot.

### Dot blot for detection of 5hmC

Dot blot analysis was carried out as previously described. Briefly, the total DNA was extracted and loaded on nitrocellulose membranes, following blocking with 10% skimmed milk for 1 h after bake at 80 °C for 10 min, then incubation with 5hmC primary antibody (1:500 dilutions, Active Motif) overnight at 4 °C. After washing the HRP-conjugated secondary antibody, the membrane was treated with ECL and scanned by a scanner. Methylene blue (MB) staining served as a loading control.

### ARE luciferase activity assay

The MDA-MB-231 cell line constructed with NQO1-ARE luciferase reporter gene maintain in our lab. In order to investigate if the stigmasterol plays a role in regulating Nrf2-ARE signal pathway, the different dose of stigmasterol was used to treat the MDA-MB-231 cells. 24 h later, the cells were harvested and lysed, the luciferase activity was determined by Dual-Luciferase Assay Kit (Promega).

### Migration and invasion assay

Ishikawa cells were seeded in the upper chamber of 24 transwell inserts with 8-um pores coated with (for invasion assay) or without Matrigel (for migration assay). The lower wells were also cultured with serum-free medium. After another 48 h culture with indicated treatment, the cells still stayed in upper champers were wiped off using a cotton swab and the invading cells on the underside of the filter were fixed and stained with crystal violet, five randomly selected fields were counted.

### Apoptosis assay

The cells underwent various treatment were harvested and fixed overnight with cold 70% ethanol. After spinning down, the cell pellet was re-suspended in PBS and incubated with Annexin V. The early apoptosis of the cells was analyzed by using flow cytometry.

### Protein and ligand structure preparation

The structure of stigmasterol was exported from the data bank of Pubchem with SDF file, following translated to Mol2 format with Open Babel, then imported into SYBYL 2.0 package for Tripos optimization. The 3D structures of Nrf2 and PDB ID (PDB ID: 2FLU, 5F72, 3WN7 and 4ZY3) of the protein were selected from Protein Data Bank (PDB) based on the antagonistic effect of the study.

### Protein–ligand docking calculations

The feasibility of stigmasterol to be ligands for Nrf2 structures was evaluated using molecular docking. This was performed using Surflex-Dock module of SYBYL to evaluate the performance of the protein–ligand docking. If the total score was greater than 5 that indicates the binding between stigmasterol and Nrf2 is good. In addition, combined with a C score, a score close to 5 is considered to have better activity.

### Identifications of residues interacting with stigmasterol on Nrf2 binding site

LigandScout 3.0 was used to identify protein residues that interact with stigmasterol with greatest affinities.

### Statistical analysis

SPSS 19.0 was used for analyzing the cellular growth, changes of western blot bands and apoptosis rate. P < 0.05 was considered significant difference when compared with control group.

## Results

### Nrf2 overexpressed in endometrial carcinoma tissue

**Fig. 1:**
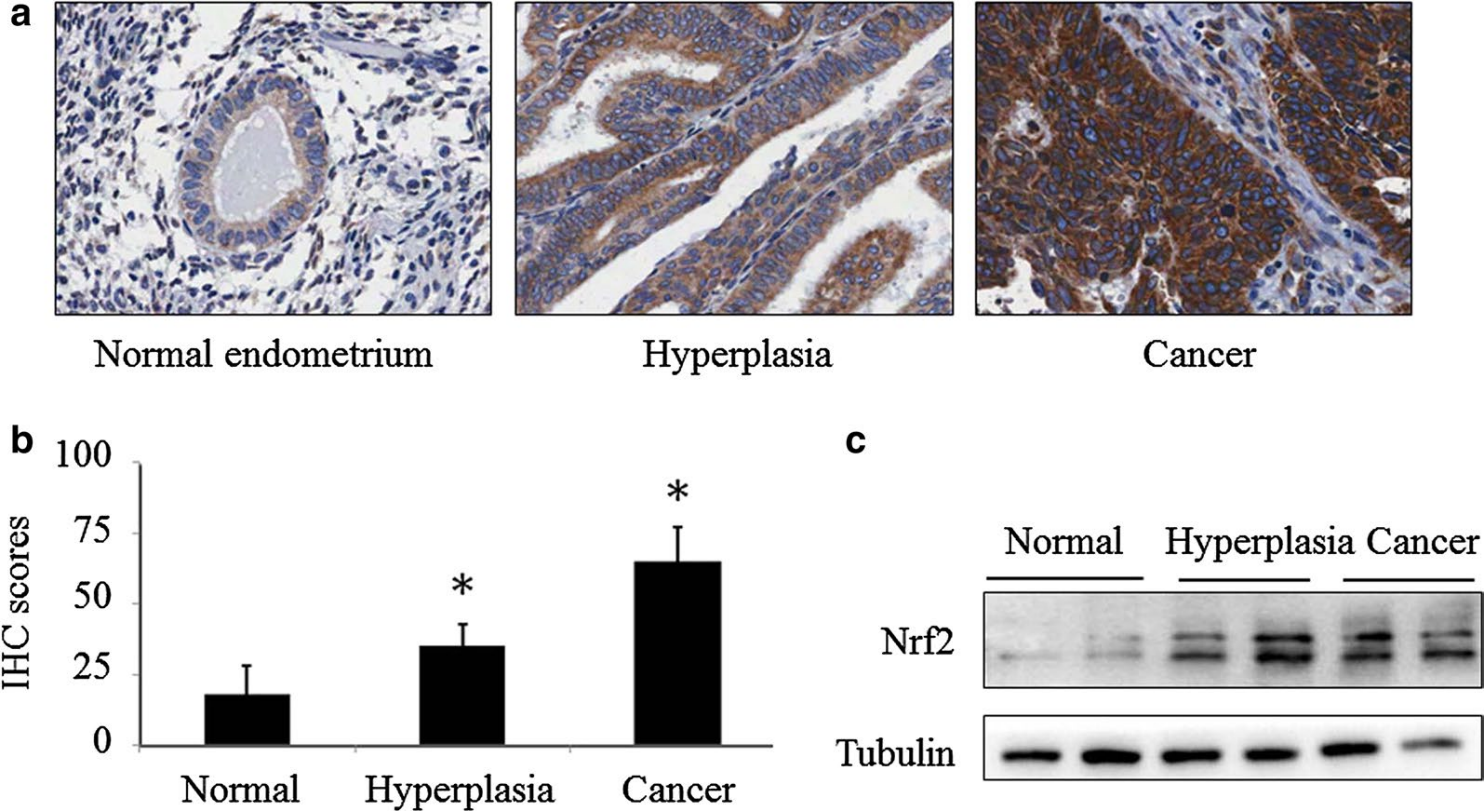
Nrf2 is overexpressed in endometrial cancer. The expression pattern of Nrf2 in normal endometrium (n = 10), hyperplasia (n = 20) and endometrial cancer (n = 90) was assessed using IHC staining. Representative images were captured at 100× magnification (**a**). The IHC scores in endometrial lesion tissues compared with normal endometrial tissues are presented (**b**). *p < 0.05 compared with normal endometrial tissues. The expression pattern of Nrf2 in endometrial fresh tissue was determined by western blot (**c**)

To identify the role of Nrf2 in endometrial cancer development, we first investigate the expression pattern of Nrf2 in healthy controls and patients with various endometrial disorders, including simple hyperplasia and cancer. It was found that the weak staining signal was present in normal endometrium, whereas, the moderate Nrf2 staining signal was shown in simple hyperplasia tissue samples, the strong staining signal appeared in endometrial cancer samples (Fig. 1a, b). Meanwhile, we collected fresh endometrial tissue with various lesions and determined the expression of Nrf2 with western blot. An increasing trend of Nrf2 expression was observed accompanying with the lesion progress from normal endometrial tissue, to hyperplasia and carcinoma tissue (Fig. 1c). These results suggest that Nrf2 play an important role in endometrial cancer development.

### Nrf2 drives chemotherapy resistance

**Fig. 2:**
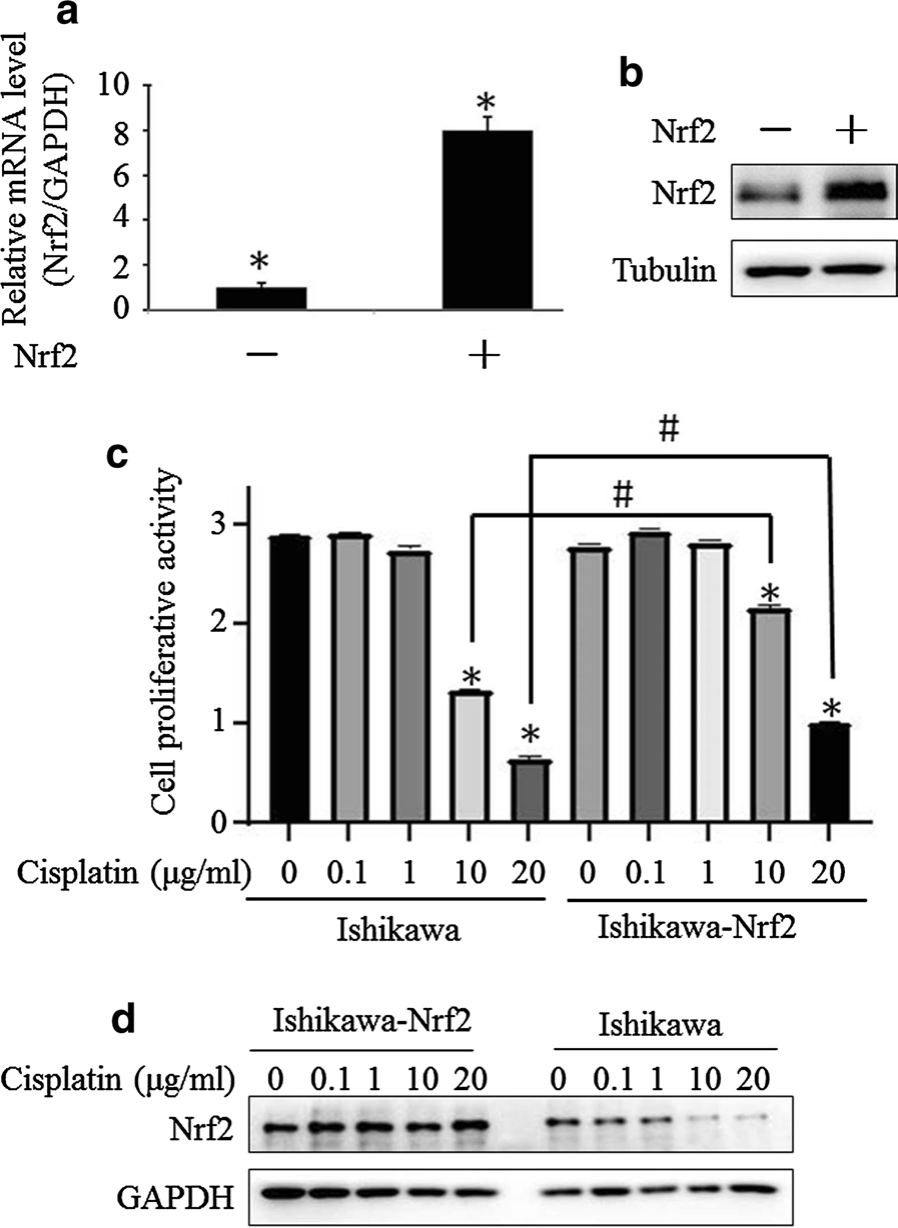
Nrf2 involves in chemotherapy resistance. Nrf2 is high expressed in Nrf2 transfected stable cell line in mRNA level (**a**) and protein level (**b**). CCK8 assay was performed to determine the inhibition effect of cisplatin on cell proliferative activity. *p < 0.05 compared with the control group (**c**). Western blot was used to detect the change of the Nrf2 protein after indicated dose of cisplatin treatment in both parental Ishikawa cells and Ishikawa-Nrf2 cells (**d**)

Chemotherapy resistance is a pivotal obstacle for endometrial cancer therapy. In order to investigate the role of Nrf2 in chemotherapy resistance, we established Nrf2 overexpressed stable cell line. As showed in Fig. 2a, the stable cell line showed an approximately eightfold increasing Nrf2 expression in mRNA level. Similarly, the increase Nrf2 protein expression also has been observed (Fig. 2b). Subsequently, we estimated the proliferative activity after cisplatin treatment in Ishikawa cells and Ishikawa-Nrf2 stable cells. It was found that cisplatin could suppress the cellular growth with a dose-dependent manner in both Ishikawa and Ishikawa-Nrf2 cells, however, the parental Ishikawa is more sensitive to cisplatin treatment (Fig. 2c). Moreover, we further investigated the expression of Nrf2 in both cell lines. We found that Nrf2 potently declined in Ishikawa cells when it exposure to increase dose of cisplatin, while, the decline is slowly in Ishikawa-Nrf2 cells (Fig. 2d). Therefore, it implies that Nrf2 plays an essential role in endometrial cancer chemotherapy resistance.

### Stigmasterol is a potential inhibitor of Nrf2

**Fig. 3:**
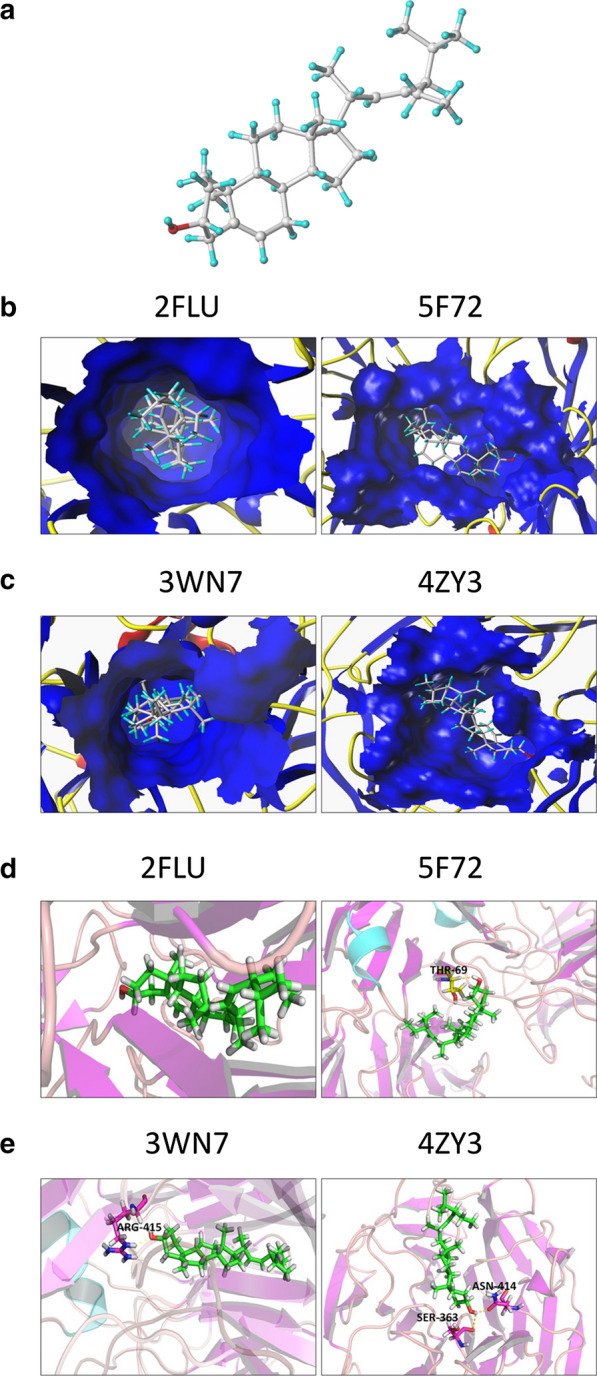
Stigmasterol is the Nrf2 inhibitor candidate. Network pharmacology screens the possible inhibitor candidate. **a** the molecular structure of stigmasterol. White represents carbon atom, blue is hydrogen atom, red is oxygen atom. The predicted human Nrf2 structural domain 2FLU and 5F72 (**b**) and mouse Nrf2 domain 3WN7 and 4ZY3 (**c**) with which stigmasterol binds. It shows the key amino acid of above structural domain interacting with stigmasterol, including human (**d**) and mouse (**e**) Nrf2 protein

Since Nrf2 involves in endometrial cancer chemotherapy resistance, it is an urgent to found a drug to inhibit this signal pathway. A potential candidate inhibitor called stigmasterol was selected by network pharmacology. The molecular structure of stigmasterol is shown in Fig. 3a. As showed in Fig. 3b, c, stigmasterol can enter into Nrf2 protein active pocket and the docking sites lie in this active pocket. A higher score indicated higher accuracy of the binding and we found these all had good binding property except 5F72 (Table 2; Additional file 1: Table S1). Yellow virtual bond is stable hydrogen bond formed between small molecule and protein. For the 5F72- stigmasterol complex, they interact with a hydrogen bond and the relevant aminoacid is Thr-69. Stigmasterol bound to the active site of 3WN7 via one hydrogen bond interaction, which were found to exist between the tested molecule and Arg-415. For 4ZY3- stigmasterol complex, which interacts through two hydrogen bonds and the key residues are Asn-414 and Ser-363 (Fig.D&E). Although the sores in mouse is higher than that in human (Table 2; Additional file 1: Table S1), considering it may exist a gap between theoretical prediction and physical truth, we decided to further verify the interactions through experiments. From above data, it suggests that stigmasterol is a potential candidate Nrf2 inhibitor.

**Table 2:** The predicted results of stigmasterol matches with Nrf2 protein

Nrf2 active structural domain	Human		Mouse	
	2FLU	5F72	3WN7	4ZY3
Total score	6.7571	3.6141	6.695	6.1729
C score	2	1	3	4

### Stigmasterol sensitizes endometrial cancer cell to cisplatin by suppression of Nrf2 expression

**Fig. 4:**
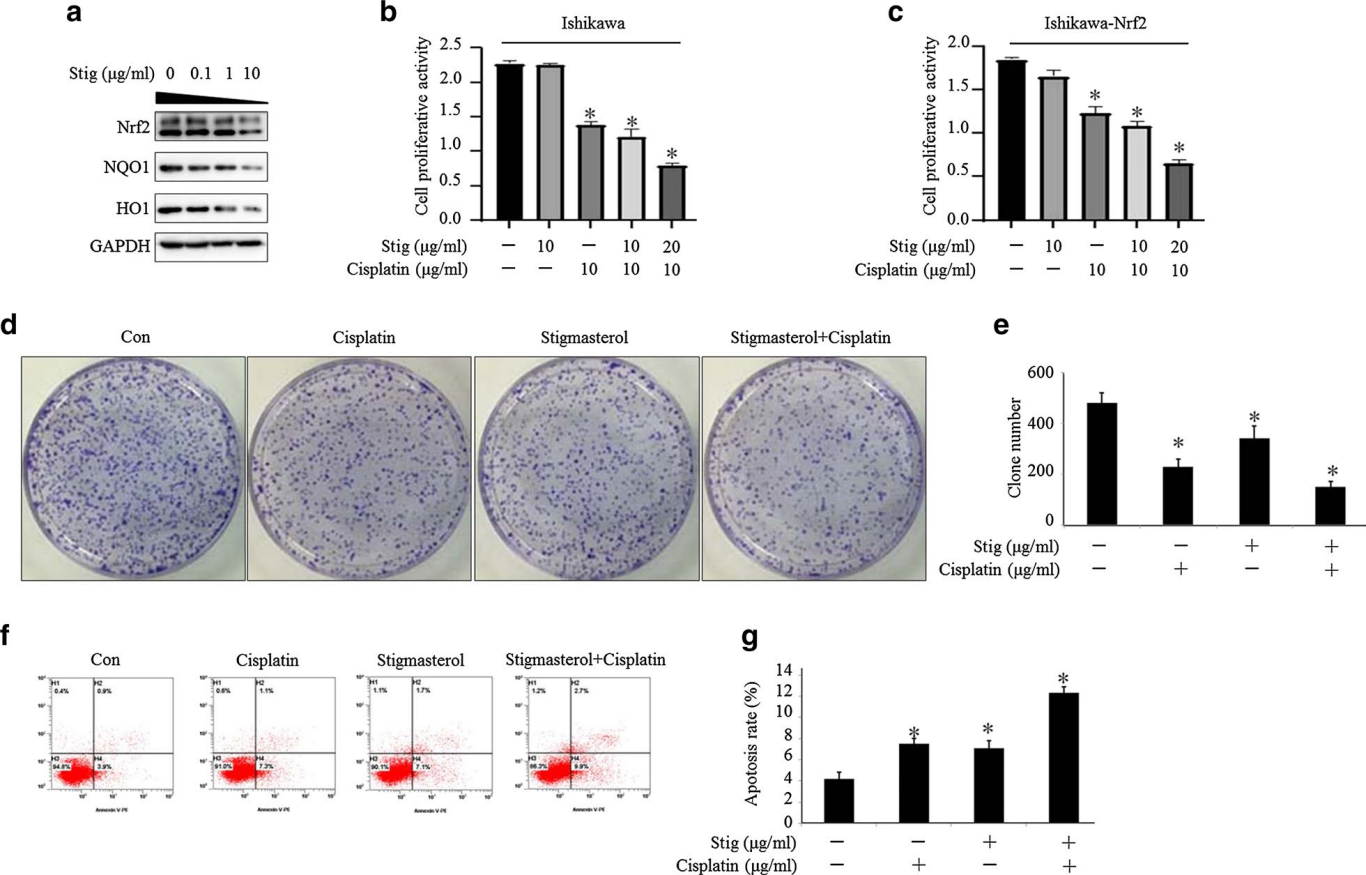
Stigmasterol sensitizes endometrial cancer cell to cisplatin by suppression of Nrf2 expression. Western blot was used to detect the effect of stigmasterol on Nrf2 expression (**a**). Stigmasterol enhances the inhibitory effect of cisplatin on EC cell proliferation. Stigmasterol was used at indicated doses; a CCK8 assay (**b**, **c**) and colony formation assay (**d**, **e**) were used to assess proliferation. The left panels show representative images of crystal violet-stained colonies. Right panels show comparison of colony numbers following indicated treatments. *p < 0.05 compared with control (**d**, **e**). The early apoptosis was determined by FCM, *p < 0.05 compared with control (**f**, **g**)

As shown in Fig. 4a, stigmasterol suppressed Nrf2, NQO1and HO1 expression with a dose dependent manner. Interesting is that there is no effect on cellular growth when stigmasterol treated Ishikawa cells alone. Cell growth assays showed that, in combination with cisplatin and stigmasterol treatment significantly inhibited the growth of endometrial cancer compared to treatment with the therapeutic drug alone (Fig. 4b). Similarly, in Nrf2-overexpressed cells, stigmasterol also could enhance the inhibition effect of cisplatin (Fig. 4c). The clonogenic assay produced a similar result; stigmasterol enhanced the inhibitory effect of cisplatin on endometrial cancer clonal growth (Fig. 4d, e). To determine whether combinational treatment with cisplatin and stigmasterol could affect the early apoptosis, the flow cytometry assay was performed. It was found that the early apoptosis rate is higher when Ishikawa cells were exposed to both stigmasterol and cisplatin compared to stigmasterol or cisplatin alone (Fig. 4f, g), which suggests that stigmasterol is a potential inhibitor for Nrf2 and plays a critical role in enhancing chemotherapy sensitivity.

### Stigmasterol enhances the inhibition effect of cisplatin on migration and invasion activity

**Fig. 5:**
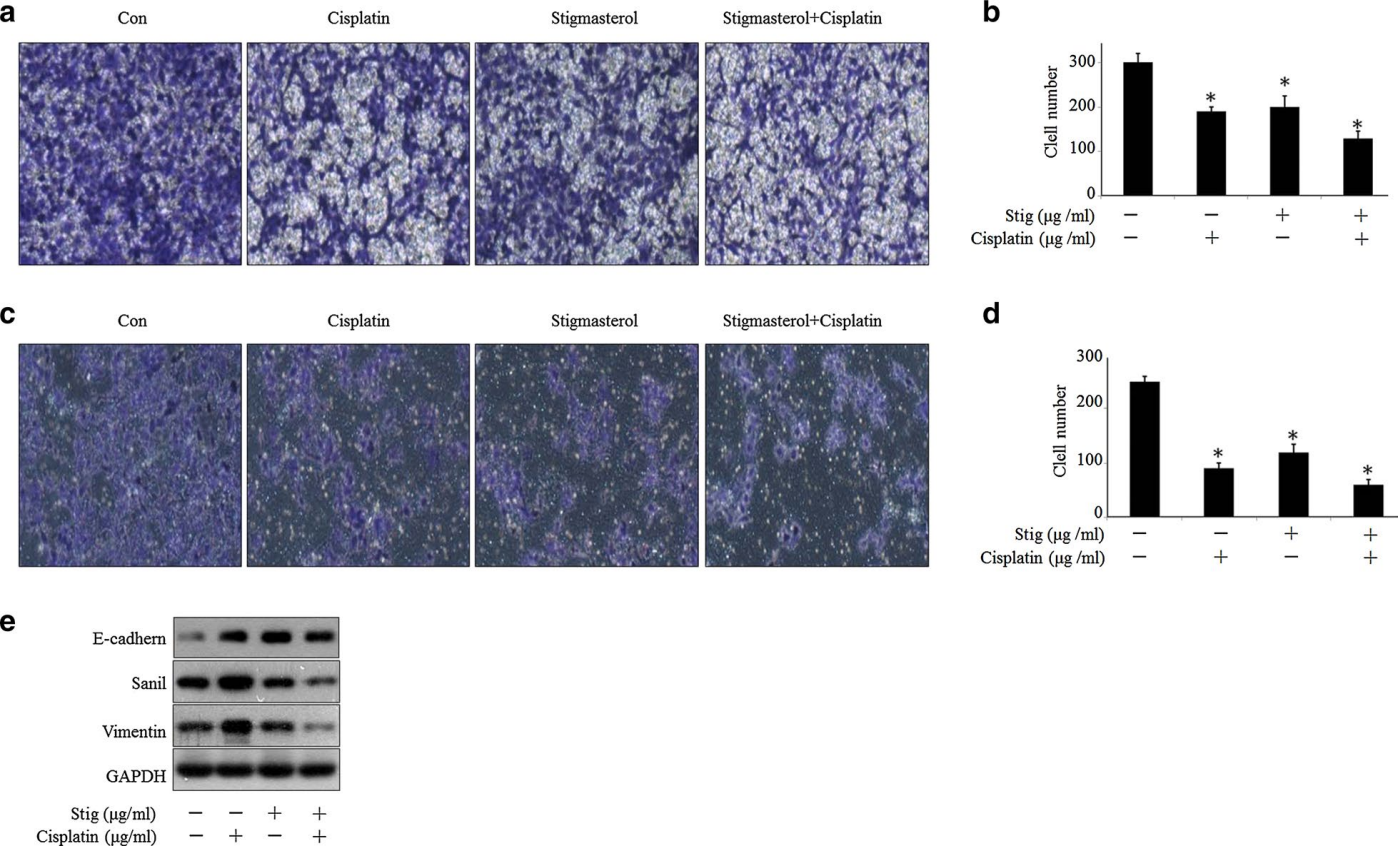
Stigmasterol enhances the inhibition effect of cisplatin on migration and invasion activity. The cells were seed on the transwell chambers on the 24-well plate, after indicated treatments, the migration cells (**a**, **b**) and invasive cells (**c**, **d**) were counted, respectively. *p < 0.05 compared with control. The migration and invasion related molecules were detected by western blot (**e**). The drug concentrations are as follows: cisplatin, 10 μg/ml/ml; stigmasterol, 10 μg/ml

As a potential inhibitor of Nrf2 signaling, we are wonder if the inhibition effect of stigmasterol could extend to other cancer malignant biological functions. Currently, it was found that stigmasterol not only facilitated cisplatin-driven migration inhibition but also invasion suppression (Fig. 5a–d). Therefore, we further estimated the expression of migration or invasion related molecules. The joint treatment with both stigmasterol and cisplatin significantly decreased snail and vimentin expression in Ishikawa cells,however, reverse expression pattern in E-cadherin has been observed (Fig. 5e).

### Stigmasterol enhances the efficacy of chemotherapeutic drugs through TET1-mediated hydroxymethylation via the Nrf2 signaling pathway

**Fig. 6:**
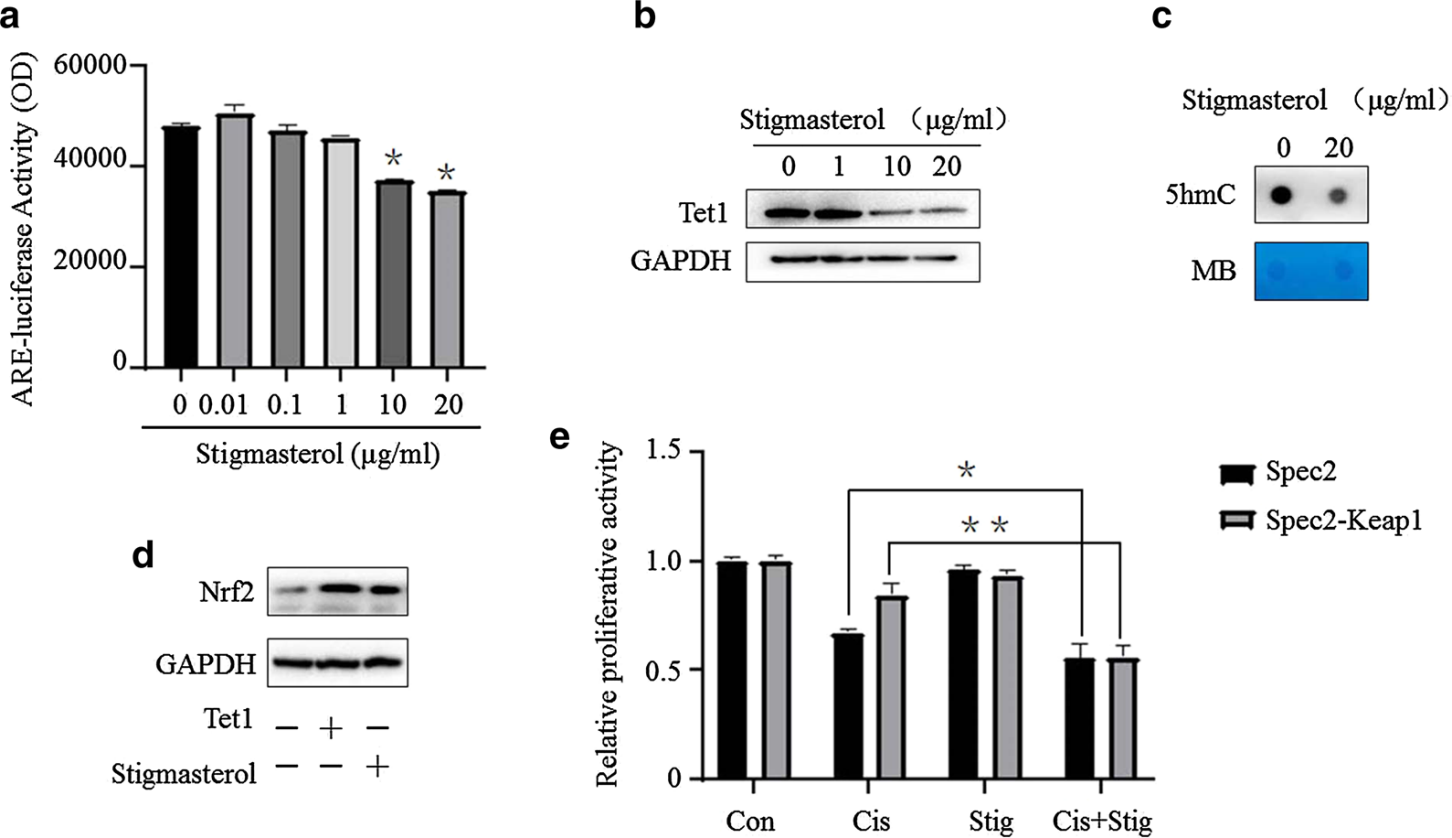
Stigmasterol enhances the efficacy of cisplatin through TET1-mediated hydroxymethylation via the Nrf2 signaling pathway. Nrf2-ARE activity assay was performed by luciferase activity determination after stigmasterol treatment with indicate dose. *p < 0.05 compared with control (**a**). Western blot was used to estimate the effect of stigmasterol on Tet1 expression (**b**). Dot-blot assay results of EC cells with stimasterol treatment (**c**). Full length of Tet1 plasmid was tranfected in Ishikawa cells, then stimasterol was used to treat these cells. Western blot was carried out to determine whether tet1 overexpression could block stigmasterol-induced Nrf2 decline (**d**). CCK-8 was used to detect the effect of stigmasterol and cisplatin on the spec-2 cells with low level of Nrf2 due to stablely transfection with Keap1, *p < 0.05, ** *p *< 0.01 compared with control (**e**)

Although stigmasterol could inhibit Nrf2 expression in both mRNA and protein levels, if the whole Nrf2 signaling and Nrf2-ARE activity have been inhibited is not clear. Currently, luciferase activity assay disclosed that stigmasterol significantly suppressed Nrf2-ARE activity (Fig. 6a). Previous study demonstrated that Nrf2 is regulated by Tet1 via hydroxymethylation. Whether stimasterol affects the Tet1-driven hydroxymethylation has not been clarified. In this study, it was found that stimasterol inhibited Tet1 with a dose manner (Fig. 6b). The dot bot demonstrated that stigmasterol obviously decreased the 5hmC level (Fig. 6c). Moreover, we found that stigmasterol could block Tet1 induced Nrf2 expression (Fig. 6d). These results suggest that stigmasterol could inhibit Nrf2 by suppressing Tet1-drived hydroxymethylation. In addition, we decreased Nrf2 level by transfection with keap1 plasmid and found that low level of Nrf2 resulted in enhanced sensitive to cisplatin (Fig. 6e).

## Discussion

Although many efforts and progresses in endometrial cancer therapy have been observed in the past, the 5 year survive rate has not been significantly improved [10, 19, 27, 28]. The high failure rate responding to chemotherapy in endometrial carcinoma attributes to the unclear molecular mechanisms of chemoresistance and there is no effective therapeutic strategy for overcoming the resistance. Therefore, it is urgent to discover the molecular mechanism and further find the potential candidate small molecule compound to facilitate the chemotherapy sensitivity.

In previous study, it has been demonstrated that chemoresistance is associated with the crosstalk between leptin-induced Notch and IL-1 signaling in endometrial cancer. At the most recent, a study pointed that cancer stem cells are responsible for cancer proliferation and chemoresistance [20], it was found that SPARC-related modular calcium binding 2 (SMOC-2) drive the endometrial cancer chemoresistance functional as a endometrial cancer stem cell molecule. Similarly, Saygin et al. reported that CD55, as unique signaling node, drives self-renewal and chemoresistance in endometrioid cancers [29]. Other study found that autophagy also involves in chemoresistance [30]. Despite these progresses have been achieved, the 5-years survival rate of those patients with endometrial cancer has not been remarkably improved. Thus, we carried out serial studies focusing on this issue. We found that IDH1 is overexpressed in endometrial cancer tissue and plays an essential role in chemoresistance. Moreover, we observed that chemoresistance was enhanced via a regulatory loop in which Nrf2 activated IDH1-ɑ-KG-TET1-Nrf2 signaling via binding to the ARE sites in the IDH1 promoter region [22]. This study highlights a critical role of IDH1-ɑ-KG-TET1-Nrf2 signaling in chemoresistance and suggests that rational combination therapy with metformin and chemotherapeutics has the potential to suppress chemoresistance. In addition, we also found that Nrf2 play an essential role in endometrial cancer development and chemoresistance. However, we did not further demonstrate how Nrf2 involves in chemoresistance and did not search for a potential medicine targeting Nrf2 for cancer therapy.

Increasing evidence from recent studies showed that the transcription factor NF-E2-related factor 2 (Nrf2) plays a critical role in promoting cancer recurrence through increased tolerance to adjuvant chemo- and/or radiation therapies. It has been well addressed that Nrf2 involves in cancer chemoresistance [11, 19]. The mechanisms of Nrf2 mediated drug resistance involve multiple genes and details of the molecular pathways of such drug resistance have been summarized elsewhere. Most recent, it has been reported that Nrf2 contributed to chemoresistance and was associated with a poor prognosis in pancreatic cancer patients. Wu et al. also reported that Nrf2 induced cisplatin resistance via suppressing the iron export related gene SLC40A1 in ovarian cancer cells [31]. In our previous endometrial cancer studies, we showed that high level of Nrf2 expression is clearly responsible for chemoresistance [19]. More importantly, brusatol, a specific inhibitor of Nrf2, could reverse chemoresistance in multiple cancers including endometrial cancer, but the detail mechanism needs further study. In current study, it was found that Nrf2 is aberrantly expressed in endometrial cancer or pre-cancer tissues (Fig. 1A). Stably transfection of Nrf2 resulted in cisplatin resistance accompanying with slightly decline of Nrf2 protein in Ishikawa-Nrf2 cells (Fig. 2c, d). These data suggest that Nrf2 plays an essential role in chemoresistance in endometrial cancer.

Since Nrf2 involves in chemoresistance, targeting this molecule maybe enhance the response to chemotherapy. Thus, downregulating Nrf2 protein level or repressing its transcription activation is a possible way to inhibit Nrf2 and Nrf2-dirven chemoresistance. Previous study showed that brusatol could reverse chemoresistance in several different kinds of cancer by downregulation of Nrf2 expression [21, 32, 33]. However,further study found that brusatol is not directly repress the Nrf2 pathway, it has broad-spectrum inhibition effect on the majority of detected proteins. So, this nonselective mechanism of brusatol limits its possibility of application in clinical practice. Since then, increasing potential candidate inhibitor of Nrf2 has been reported, including glucocorticoids, flavonoids, wogonin, dexamethasone, and luteolin. However, these compounds have relative weak inhibition effect on Nrf2 expression compared with brusatol. In current study, we found a possible inhibitor of Nrf2 by model analysis via network pharmacology. It is stigmasterol which distributes in many plants such as halodrymium.

In this prediction model, stigmasterol matches more with mouse Nrf2 protein, rather the human Nrf2 protein (Fig. 3). So, we compared the gene sequence of Nrf2 between mouse and human, it was found it is a highly conservative gene. This prompts us further investigate whether stigmasterol could repress human Nrf2 expression, especially in human endometrial cancer cells. As expected, we found that stigmasterol inhibited Nrf2 protein expression in human endometrial cancer with a dose manner. Most importantly, stigmasterol combine with cisplatin could significantly suppress Nrf2-ARE activity and enhance cisplatin-induced inhibitions on cellular growth, migration and invasion. In addition, stigmasterol enhanced cisplatin-induced early apoptosis. In previous study, most inhibitors of Nrf2 repress Nrf2 protein level through suppressing its transcription activation except of brusatol [14, 15, 17]. Here, we found that stigmasterol inhibited Tet1 expression and Tet1-induced hydroxymethylation, following in turn inhibit Nrf2 protein expression. So, the decline of Nrf2 protein by stigmasterol also attributes to decrease activity of transcription.

A complete understanding of the role of Nrf2 will facilitate overcoming chemoresistance. The joint effects between stigmasterol and cisplatin observed in endometrial cancer cells provide the possibility of achieving improved therapeutic effects with lower chemotherapeutic doses. Our findings provide the foundation for the hypothesis that stigmasterol sensitizes endometrial cancer cells to chemotherapy by suppression of Nrf2 expression.

## Conclusion

Our results showed that Nrf2 plays an essential role in chemoresistance in endometrial cancer. Moreover, stigmasterol, as a novel potential inhibitor of Nrf2 has been identified, has the potential to suppress chemoresistance in combination therapy with cisplatin.

## Supplementary information

**Additional file 1: Table S1.** Docking details for stigmasterol on four Nrf2 active structural domains.

## Data Availability

Data files generated and/or analyzed during the current study are available from the corresponding author upon reasonable request.

## Abbreviations

5hmc: 5-hydroxymethylcytosine
ARE: Antioxidant response element
IDH1: Isocitrate dehydrogenase [NADP]
IHC: Immunohistochemical
NQO1: NAD(P)H dehydrogenase [quinone] 1
NRF2: Nuclear factor erythroid 2-related factor 2
PBS: phosphate buffer saline
PCR: Polymerase chain reaction
TET1: Tet methylcytosine dioxygenase 1
